# Acute Hemiballismus as the Presenting Feature of Parietal Lobe Infarction

**DOI:** 10.7759/cureus.4675

**Published:** 2019-05-16

**Authors:** Avani R Patel, Amar R Patel, Soaham Desai

**Affiliations:** 1 Internal Medicine, Northern California Kaiser Permanente, Fremont, USA; 2 Neurology, Pramukhswami Medical College, Karamsad, IND

**Keywords:** stroke, hemiballismus, parietal lobe, subthalamic nucleus, basal ganglia, hemichorea, tetrabenazine, hemichorea-hemiballismus syndrome

## Abstract

It is widely believed that hemiballismus and chorea are suggestive of a basal ganglia subthalamic nucleus lesion; however, this not a rule. We report the case of a 63-year-old male with complaints of slurred speech, increased movement of the left half of his body, and headache. He had diabetes, hypertension, and a past medical history of stroke with residual weakness over the right side of his body. The patient developed the sudden onset of irregular, large amplitude, increased involuntary movements of his left upper and lower limbs with a flinging pattern. His blood sugar and serum osmolality were normal. His magnetic resonance imaging (MRI) showed an acute right parietal lobe infarction. Patients can experience hemiballismus with lesions other than the subthalamic nucleus in the basal ganglia. This is contrary to the classic belief that hemiballismus is associated with, and only with, lesions in the subthalamic nucleus. This manuscript describes a case of hemiballismus occurring in a patient secondary to a parietal lobe infarction.

## Introduction

Hemiballismus is defined as irregular, involuntary, large amplitude flinging movements by the limbs confined to one side of the body [1]. Depending on the areas of the brain affected, patients can present with certain common clinical features as per the affected area. For example, if a patient has an infarction of the motor cortex, then they commonly present with hemiplegia or paralysis on one side of the body. Infarctions involving language areas of the brain will present with aphasia or impairment of language. This will affect the production or comprehension of speech and the ability to read and write.

Hemiballismus was first described in 1949 [2]. Dr. J.R. Whittier, who was studying the subthalamic nucleus of rhesus monkeys at the time, noted that when there was a minimum of 20% area damage, then hemiballismus would be induced in the contralateral limbs [2]. The traditional belief of hemiballismus being a direct result of subthalamic nucleus infarction originates from his work. The following report is one of the cases that disprove that traditional belief.

## Case presentation

A 63-year-old male was admitted with complaints of acute onset, increased, involuntary, violent movements of his left upper and lower limbs. His past medical history included diabetes mellitus, hypertension, and an ischemic stroke 20 years prior with residual right spastic hemiplegia. He had a below-the-knee amputation done on the right lower limb three years earlier due to peripheral vascular disease and gangrene.

After the patient was admitted, a full history and physical examination were performed. With the exception of the amputated right lower limb, the examination of his vitals, head, neck, cardiovascular, pulmonary, and abdominal systems were without abnormalities.

On neurological examination, the patient was alert, awake, and oriented to time, place, and person. He was obeying vocal commands but had continuous, large amplitude, irregular, involuntary movements in his left upper and lower limbs. The movements had a flinging pattern suggestive of hemiballismus (see Video 1). His muscle power was decreased on the right side of his body with normal muscle power in both the left upper and lower limbs. No paralysis was noted over the left side of the body.

**Video 1:** Hemiballismus Seen in the Patient Secondary to Right-sided Parietal Lobe Infarction The patient was unable to prevent the large amplitude flinging movements suggestive of left-sided hemiballismus. The video was created within 48 hours of the patient's stroke.

**Figure 1:**
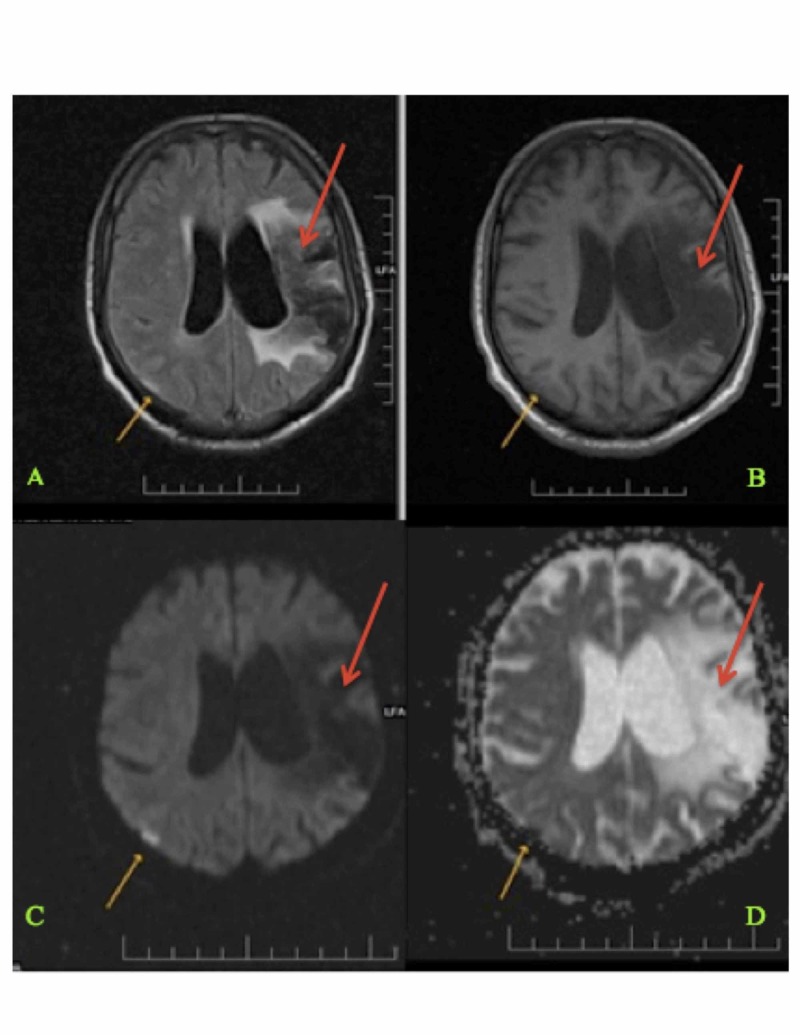
Magnetic Resonance Imaging (MRI) of the Brain Showing Current and Previous Infarction Sites (Panels A-D) Magnetic resonance imaging of the brain showing an acute right-sided parietal lobe infarct (shown with yellow arrows) and an old left-sided middle cerebral artery territory infarct (shown with red arrows).

His blood sugar, serum electrolytes, and serum osmolality were within the normal range. Magnetic resonance imaging (MRI) of the brain revealed an acute right parietal lobe infarct with an old middle cerebral artery territory infarct (Figure 1). 

## Discussion

Hemiballismus is a series of abnormal flinging movements that are involuntary and involve one-half of the body [1]. Depending on the side infarcted, there is hemiballismus of the contralateral side. Hemiballismus usually involves an arm or a leg, is proximal in most cases, and can have facial involvement in approximately half of the patient cases [3]. Another hyperkinetic movement disorder confined to one side of the body is hemichorea. It is an involuntary, occasional, rapid, non-patterned series of movements. Hemiballismus is considered to be a more severe expression of hemichorea [3]. Hemichorea-hemiballismus (HCHB) syndrome is a condition where patients have both of these movements in combination with one another [1]. The commonly described causes of HCHB syndrome include hemorrhagic or ischemic stroke, neoplasm, systemic lupus erythematosus, a hyperglycemic hyperosmolar non-ketotic (HONK) state, Wilson's disease, and thyrotoxicosis [4]. Even in these cases, MRI of the brain usually reveals T2 fluid-attenuated inversion recovery (FLAIR) hyperintense signals involving the basal ganglia, more specifically, the part that contains the subthalamic nucleus. One documented case had a cerebral cavernous angioma in the posterior limb of the left internal capsule and in the lateral part of the left thalamoganglionic region [5]. The common belief is that hemiballismus is associated with a subthalamic nucleus dysfunction. However, with the advent of MRI and its increased use in acute stroke care, an increasing number of hemiballismus cases are proven to have confirmed parietal lobe involvement.

**Figure 2:**
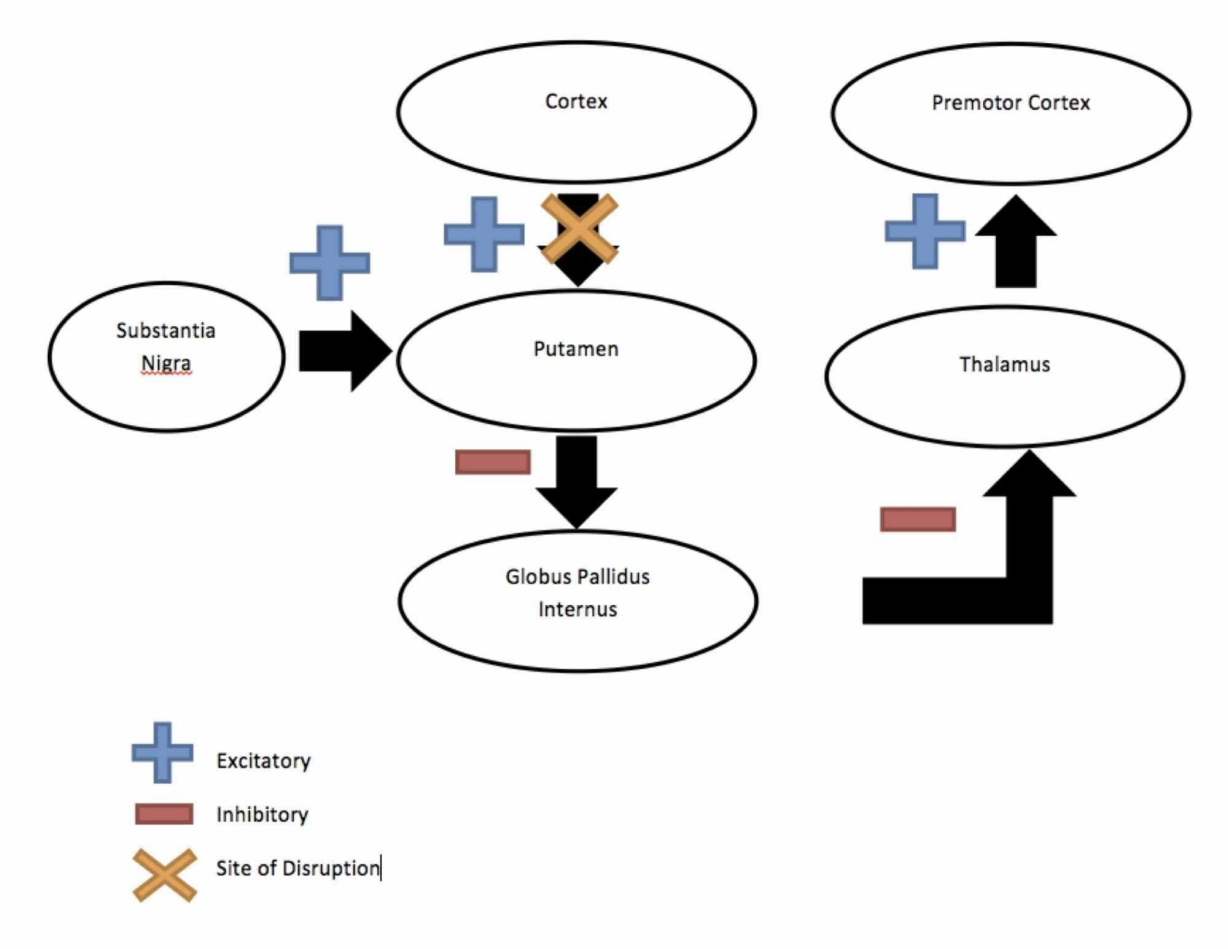
Direct Pathway (in the Case of Cortical Hemiballismus) Demonstrating the direct pathway from the cortex to the caudate to the internal palladium, thus accounting for dyskinesias seen in cortical hemiballismus

**Figure 3:**
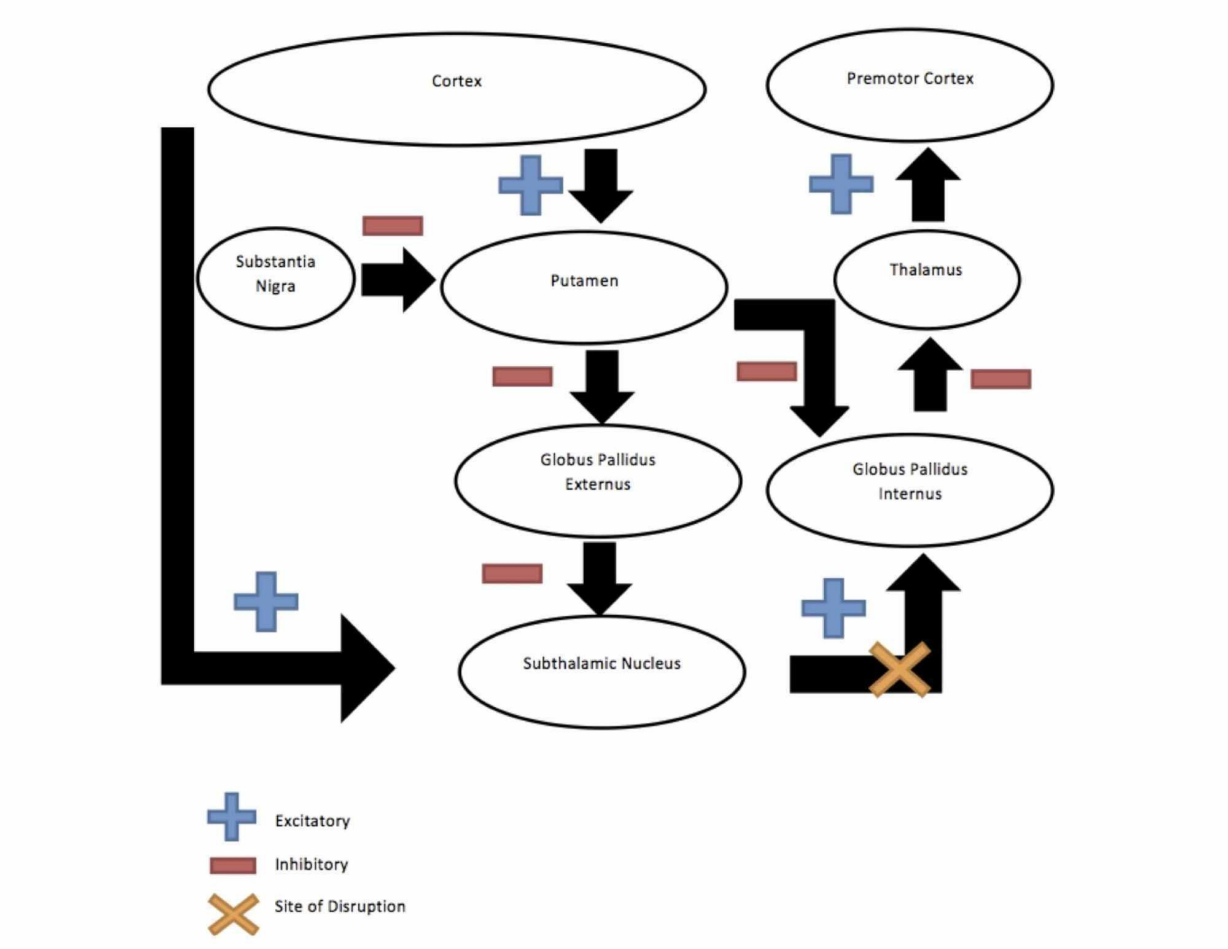
Indirect Pathway (in the Case of Subthalamic Hemiballismus) Demonstrating the indirect pathway, going from the cortex to the caudate nucleus to the external palladium to the subthalamic nucleus and finally to the internal palladium. This accounts for akinesia in the case of subthalamic hemiballismus.

We describe a patient who had acute hemiballismus following an ischemic stroke involving the parietal lobe and not the basal ganglia or, more specifically, the subthalamic nucleus. A magnetic resonance angiogram (MRA) of the intracranial vessels was also found to be normal. There have been previous cases of hemiballismus reported with parietal lobe infarct with the absence of basal ganglia infarction (Table 1). The earliest published case of hemiballismus with parietal infarct was in 1997 [6]. There exists a balance between the direct pathway (cortex-caudate-internal pallidum, accounting for the dyskinesias) and the indirect pathway (cortex-caudate-external pallidum-subthalamic nucleus-internal pallidum, accounting for the akinesia), which modulate the thalamocortical retroactive pathway for control of voluntary movements [7]. We generated diagrams of the direct pathway and an indirect pathway below for clarification (Figures 2-3). Classically, lesions of the subthalamic nucleus are said to disrupt this balance but that does not exclude lesions of the corticostriatal fibers. Under normal physiologic conditions, the cerebral cortex provides excitatory stimuli to the basal ganglia [8]. Thus, lesions involving corticostriatal fibers (in the parietal cortex) may disrupt the balance of basal ganglia circuits [9]. This is one possible hypothesis for the occurrence of hemiballismus in patients with parietal lesions. The other possible hypothesis could be associated hypoperfusion of the subthalamic region not picked up on the initial MRI in the hyperacute stage of the infarct. In 1989, Barinagarrementeria et al. described a case of parietal lobe infarct where perfusion studies revealed evidence of reduced perfusion to the radiologically unaffected striatum [6, 10-11]. They suggested that hypoperfusion of the subthalamic region undetected on the initial scan could be the underlying influence in “cortical” hemiballismus in their case. This is possible because the subthalamic nucleus is located at the arterial border zone position between the anterior and posterior circulation of which compromising carotid stenosis could lead to hypoperfusion [12]. In our patient, special imaging techniques for detection of hypoperfusion were not required because the first imaging itself was done more than 24 hours post-symptom onset and also angiography findings were normal; however, the possibility of subthalamic hypoperfusion cannot be entirely ruled out.

**Table 1:** Cases of Hemiballismus-Hemichorea Occurring with Parietal Infarction without Lesion to Basal Ganglia These are previous cases of hemiballismus-hemichorea occurring with parietal lobe infarction. In each case, there is an absence of infarction in the basal ganglia.

Author and Year	Case Report Name	Age	Sex	Duration	Side of Infarct	Treatment	Outcome
1997, Mizushima et al. [6]	A Case of Hemichorea-Hemiballism Associated with Parietal Lobe Infarction	80	Male	13 days	Right-sided parietal lobe infarction was present.	Not specified	Symptoms subsided and the patient was discharged with slightly diminished deep sensation.
2003, Rosetti et al. [9]	Neurogenic Pain and Abnormal Movements Contralateral to an Anterior Parietal Artery Stroke	74	Male	3 weeks	Acute infarction of the right anterior parietal cortex, extending to the upper posterior temporal lobe and the adjacent white matter	Haloperidol and anticoagulants	Hemiballismus subsided
2004, Al-Yacoub et al. [13]	Hemiballismus from a Parietal Stroke in a Parkinson Patient	77	Male	4 days	Large right-sided parietal infarct was present.	Very low dose clozapine	Hemiballismus reduced
2006, Sugiura A, Fujimoto M [14]	Facial Chorea and Hemichorea due to Cardiogenic Cerebral Embolism in the Cortex and Subcortical White matter	62	Male	1 day	Acute cortical and subcortical infarctions at the right insula, frontal, temporal, and parietal lobes.	Tiapride hydrochloride	Chorea subsided
2012, Umeh et al. [15]	Dual Treatment of Hemichorea Hemiballismus Syndrome with Tetrabenazine and Chemodenervation	65	Male	9 months	Right posterior frontal lobe white matter and small cortical infarcts in the right temporal-frontal-parietal junction	Haloperidol, risperidone, valproic acid, tetrabenazine, onabotulinum toxin A	Moderate reduction of symptoms
2013, Hwang et al. [16]	Cortical Hemichorea-Hemiballismus	70	Female	2 months	Left parietal cortex	Haloperidol	Follow-up after four years confirmed no further episodes.
2015, Shrestha et al. [1]	Hemiballism in a Patient with Parietal Lobe Infarction	61	Male	2 days	Acute posterior left parietal lobe infarction	Aspirin, atorvastatin, warfarin	Movements resolved spontaneously.

The majority of the cases of hemiballismus have a favorable outcome by either spontaneous resolution or by the improvement of movements within weeks to months. During the early stages, hemiballismus can be injurious to the patient and would require prompt medical treatment. Medications used to treat hemiballismus include neuroleptics, valproic acid, topiramate, gabapentin, and dopamine-depleting drugs. Patients with chronic hemiballismus, refractory to medical treatment, may need a pallidotomy, a thalamotomy, or deep brain stimulation. We prescribed tetrabenazine in a dose of 12.5 mg given three times a day that was then titrated to 25 mg given three times a day during the course of a week, which led to a complete recovery of the hemiballismus in our patient. His treatment was continued for two additional weeks and was then stopped. Tetrabenazine has also been used in other cases of hemiballismus with a successful resolution of symptoms [17]. Other published cases of hemiballismus with parietal lesions have all had a good prognosis with some resolving spontaneously and others treated with neuroleptics, such as haloperidol or clozapine. None of the cases were refractory to medical treatment. 

## Conclusions

We describe a patient with a right-sided parietal lobe infarction presenting with left-sided hemiballismus that resolved after completing treatment with tetrabenazine. Our case highlights the fact that besides the subthalamic nucleus, any lesions to the cortex, to the subthalamic-pallidal fibers, to the striatopallidal connections, or to the corona radiata may induce hemiballismus in a patient. 
